# N/S Co-Doped Carbon-Coated Micro-Expanded Graphite for High-Performance Lithium-Ion Battery Anodes

**DOI:** 10.3390/ma18112477

**Published:** 2025-05-25

**Authors:** Wenjie Wang, Xuan Zhang, Xianchao Wang, Chengwei Gao, Jinling Yin, Qing Wen, Guiling Wang

**Affiliations:** 1 Key Laboratory of Superlight Materials and Surface Technology of Ministry of Education, College of Materials Science and Chemical Engineering, Harbin Engineering University, Harbin 150001, China; wangwenjie914918@163.com (W.W.); yinjinling@hrbeu.edu.cn (J.Y.); wangguiling@hrbeu.edu.cn (G.W.); 2 Heilongjiang Hachuan Carbon Materials Technology Co., Ltd., Jixi 158100, China; 3 CNBM Graphite New Material Co., Ltd., Beijing 100089, China; zhangxuan@cnbm.com.cn (X.Z.); wangxianchao@cnbm.com.cn (X.W.); 4 Jixi Quality Supervision Inspection and Testing Center of Graphite Products (National Quality Inspection and Testing Center of Graphite Products (Heilongjiang)), Jixi 158100, China

**Keywords:** natural graphite, micro-expanded graphite, N/S co-doping, biochemical fulvic acid, high-performance lithium-ion batteries

## Abstract

Natural graphite (NG) is abundant and has a high capacity for lithium-ion storage, but its narrow interlayer spacing and poor cyclic stability limit its use in high-performance lithium-ion batteries (LIBs). To address this, a N/S co-doped micro-expanded graphite composite (BFAC@MEG) was prepared by coating micro-expanded graphite (MEG) with N/S-containing amorphous carbon derived from biochemical fulvic acid (BFAC). This enhanced the electrochemical kinetics of lithium ions, improving charge transfer rates and reducing diffusion resistance. GITT results showed a higher Li^+^ diffusion coefficient than MEG and spherical graphite (SG). BFAC@MEG exhibited excellent rate performance, robust storage capacity and remarkable cycling stability. It had a specific capacity of 333 mAh g^−1^ at 1 C, 205 mAh g^−1^ at 3 C, and retained 81.57% capacity after 500 cycles. Even at 5 C, BFAC@MEG exhibits a high reversible capacity of 98 mAh g^−1^ after 200 cycles. After cycling, SEM and XPS analyses revealed a low expansion rate of 15.96% cross-sectional expansion after 300 cycles at 3 C and a stable solid electrolyte interphase (SEI) film rich in LiF and Li_2_CO_3_.

## 1. Introduction

For an extended period of time, the widespread reliance on conventional energy sources and the improper release of by-products have resulted in energy scarcity and environmental degradation [1]. As a result, it is essential to actively promote reforms in the energy sector and develop efficient and clean new energy systems. Lithium-ion batteries (LIBs) possess excellent stability, high energy density, low cost, and environmentally friendly characteristics, making them particularly popular in the realms of energy and power batteries [2,3]. The anode material is an essential element of LIBs. Over the years, scientists have been committed to exploring new anode materials. For example, there are silicon-based anode materials, fibrous phosphorus anode materials, tin-based anode materials, biomass-derived biochar (BC) anode materials, and two-dimensional covalent organic framework anode materials [4,5,6,7,8]. Nevertheless, graphite remains the primary anode material used in modern LIBs [9]. Graphite is sorted into two main types based on its formation process: artificial and natural graphite [10]. However, the cost of producing artificial graphite is substantial, and the process of graphitization requires high temperatures, making it difficult to achieve a low-carbon and environmentally friendly new energy model. China possesses a vast amount of natural graphite (NG) deposits [11]. NG possesses a layered structure that is well-suited for the intercalation/deintercalation of Li^+^. It exhibits a stable and low lithiation/delithiation potential, a high theoretical specific capacity (372 mAh g^−1^), and is environmentally friendly, making it an excellent choice for the anode in LIBs [10]. However, using NG in high-rate LIBs continues to pose a challenge.

Spherical graphite (SG) is produced through the spheronization process of natural flake graphite (FG). Compared to FG, SG exhibits a consistent particle size, a reduced specific surface area (SSA), and the advantage of isotropy [12]. At the electrode level, these advantages make SG more suitable for the anode of LIBs [13]. However, SG still has some shortcomings. SG has a narrow interlayer spacing and the limited channels hinder the rapid intercalation/deintercalation of Li^+^, resulting in slow charge–discharge rates in the battery [14]. Especially during high-current charging/discharging processes, the capacity decay of SG is particularly severe [15]. These drawbacks are not conducive to the application of SG in high-rate LIBs. In recent years, research on micro-expanded graphite (MEG) has gained significant traction. Adjusting the spacing between layers of graphite can enhance its electrochemical properties [16,17]. Li et al. [18] modified the interlayer spacing of MEG by regulating the quantity of oxidant (KMnO_4_) through an enhanced Hummers method, facilitating K^+^ transport. Lin et al. [19] synthesized MEG, utilizing HClO_4_ as an intercalating agent, and the anode exhibited excellent rate and cycling performance.

However, the slightly expanded structure will inevitably increase the SSA, leading to greater electrolyte consumption during the initial charge–discharge cycle, thereby reducing the initial coulombic efficiency (ICE) [20]. Carbon coating can effectively address this issue. It creates a protective layer on the surface of MEG, minimizing direct interaction between MEG and the electrolyte [20]. Additionally, using a carbon coating can reduce the volumetric expansion of MEG during prolonged charge–discharge cycles, thereby enhancing the lifespan of the battery [21]. As an example, Zheng et al. [22] combined MEG with pitch to produce spherical composite materials, resulting in the ICE of the battery reaching a value as high as 78%. Moreover, the introduction of heteroatoms enhances the stability and conductivity of electrode materials, thereby improving the reliability of battery performance at high rates [23,24,25]. For example, Zhuo et al. [24] uniformly coated microcrystalline graphite surfaces with amorphous carbon-containing N using a mixing carbonization strategy. Nitrogen doping enhances the material’s electrical conductivity, facilitating rapid K^+^ storage. Similarly, the co-doping of two elements can enhance the comprehensive performance of the battery [26]. Shi et al. [27] utilized N/P co-doped amorphous carbon to coat NG, and the resultant composite electrode demonstrated a specific capacity at the first discharge of 430 mAh g^−1^ and an ICE of 87.39% at 0.1 C. Yang et al. [28] developed carbon nanosheets co-doped with N and S, which exhibited exceptional electrochemical performance. Therefore, incorporating N and S into micro-expanded graphite composite materials and examining their effects on the performance of LIBs is highly significant.

Herein, a low-cost method is employed to prepare MEG, which is then modified using N- and S-rich amorphous carbon derived from biochemical fulvic acid (BFAC) through a straightforward coating process. The initial slight expansion offers an accessible and generous conduit for Li^+^ to move in and out of the material, and the BFAC coating renders the MEG surface smoother. Doping N and S into the material structure greatly lowered the charge transfer resistance and boosted the Li^+^ intercalation/deintercalation capacity in BFAC@MEG, enhancing its high-rate performance. The findings indicate that BFAC@MEG exhibits superior rate performance, enhanced lithium-ion transport efficiency, and increased stability during cycling. The Li^+^ diffusion rate of BFAC@MEG was observed to be higher than that of MEG and SG. The material exhibits enhanced reversible specific capacities, achieving 206 mAh g^−1^ at 3 C and 125 mAh g^−1^ at 5 C. At 1 C, BFAC@MEG maintained 319 mAh g^−1^ with 95.92% capacity retention after 500 cycles. At 3 C, it retained 81.57% capacity after 500 cycles, and at 5 C, it kept a reversible capacity of 98 mAh g^−1^ after 200 cycles.

## 2. Materials and Methods

### 2.1. Chemical Reagents

Spherical graphite (SG, purity > 99.95%) was sourced from China National Building Material Group Heilongjiang Graphite New Material Co., Ltd., Jixi, China. HCl and H_2_SO_4_ (purity 95.0–98.0%) were from Xilong Chemical Co., Ltd., Shantou, China. KMnO_4_ and NaNO_3_ were purchased with analytical purity from Liaoning Quanrui Reagent Co., Ltd., Jinzhou, China and Tianjin BeiLian Fine Chemicals Development Co., Ltd., Tianjin, China, respectively. Anhydrous ethanol (analytical purity) was from Chengdu KeLong Chemical Co., Ltd., Chengdu, China. H_2_O_2_ (30 wt%) and biochemical fulvic acid (BFA, reagent purity 85%) were obtained from Aladdin. H_2_O was prepared in the laboratory.

### 2.2. Synthesis Process

#### 2.2.1. Preparation of Micro-Expanded Graphite (MEG)

First, 1.0 g natural spherical graphite was combined with 0.5 g of NaNO_3_ to form a uniform mixture. Then, 12 mL H_2_SO_4_ and 0.1 g KMnO_4_ were added under the condition of an ice-water bath at −10 °C. After 5 min of stirring, the mixture was then warmed to 35 °C. The reactants were subjected to further stirring for 5 min. Following this, the solution was placed back into the ice-water bath, and 23 mL H_2_O was added to the mixture. Subsequently, the mixture was placed in a water bath heated to 98 °C and stirred continuously for 5 min. In the final step, 65 mL H_2_O, 5 mL 30 wt% H_2_O_2_, and 50 mL 10 wt% HCl were sequentially added to the reaction mixture.

The mixture underwent several rounds of filtration until it reached a neutral pH. The precipitate, after being filtered, was exposed to a drying regimen at 80 °C over 12 h to attain complete dryness. The prepared mixture was gathered and ground to a consistent fineness before its subsequent transfer to a tubular furnace for additional processing. Under an argon atmosphere, the substance was heated to 600 °C at 5 °C min^−1^ and held for 3 h, producing micro-expanded graphite (MEG).

#### 2.2.2. Preparation of BFAC@MEG

A total of 0.04 g BFA and 0.27 g MEG were added to 50% ethanol solution and stirred until completely dissolved. The mixture was then moved to an oil bath that had been preheated to 85 °C and stirred constantly until all the solvent had evaporated. Subsequently, the solid residue was collected and transferred to an oven set at 80 °C for 4 h. In the end, the material was ground into a fine powder and underwent a carbonization coating process in a tubular furnace. The temperature was gradually increased to 800 °C at 5 °C min^−1^ under an inert Ar atmosphere, where it was maintained for 2 h. The resulting product was named BFAC@MEG.

### 2.3. Materials Characterization

The microstructure of the materials was examined using scanning electron microscopy (SEM) (Hitachi, Tokyo, Japan, Model S-450/JEM100CX-II). Prior to testing, the materials were subjected to gold sputtering, and the operating voltage was set at 8–10 kV. The crystalline structure and composition of the materials were investigated through the application of X-ray diffraction (XRD) (Rigaku, Tokyo, Japan, Model D/max-TTR III). The X-ray source employed was Cu-Kα, characterized by a wavelength of 0.154056 nm. The scanning was conducted over a range of 10° to 80° at 10° min^−1^. Raman spectroscopy was utilized to investigate the composition and the extent of graphitization changes in the materials (Horiba, Tokyo, Japan, Model LabRAM HR Evolution). The excitation source was an Ar ion laser with an incident wavelength of 532 nm, and the measurement range was 500–3000 cm^−1^. X-ray photoelectron spectroscopy (XPS) was conducted using a Thermo Fisher Escalab instrument (Thermo Fisher Scientific, Waltham, MA, USA) to analyze the elemental composition and the evolution of chemical states of the materials during electrochemical cycling. The instrument utilized a monochromatic Al Kα X-ray source (h*ν* = 1486.6 eV) with a spot size of 500 μm. High-resolution spectra were collected at a pass energy of 30 eV and a step size of 0.05 eV. The binding energy of the C 1s peak was calibrated to 284.8 eV, and peak fitting analysis was conducted using XPS PEAK41 software, in which the fitting was based on a Gaussian/Lorentzian convolution function. The SSA and pore size distribution of the materials were assessed via Brunauer–Emmett–Teller (BET) analysis, which was conducted using an automated specific surface area and pore size analyzer (Micromeritics ASAP 2020 PLUSHD88, Norcross, GA, USA). The measurement gas was nitrogen, and the temperature was maintained at 77 K. The amorphous carbon content in the materials was quantitatively measured using a Q50 thermogravimetric analyzer (TGA, TA Instruments, New Castle, DE, USA). The measurements were performed in an oxygen atmosphere, with the temperature increasing from 30 to 800 °C at 20 °C min^−1^.

### 2.4. Electrochemical Measurements

The active material, binder (PVDF), and conductive agent (Super P) were homogeneously dispersed in an NMP solvent at 8:1:1. The paste requires thorough mixing for a duration of 4 h within a moisture-free environment. In the half-cell experiments, the anode and cathode materials were pure lithium and a graphite-based material, respectively. The quantity of active material loaded on the anode was 1.5 mg cm^−2^. Copper foil, which serves as the current collector, is evenly coated with the paste and then placed in a vacuum drying chamber for an extended duration, usually overnight. Batteries were assembled in a glove box from Shanghai Company Ltd.,(Shanghai, China). (O_2_ < 0.1 ppm, H_2_O < 0.1 ppm). The electrolyte used for the battery is a lithium-ion secondary electrolyte (LB-002, 1M LiPF_6_, DMC:EC:EMC = 1:1:1 vol%). The testing procedures encompassed galvanostatic intermittent titration technique (GITT) and galvanostatic charge–discharge (GCD), which were conducted using a CT-4008 Neware system. Additionally, cyclic voltammetry (CV) and electrochemical impedance spectroscopy (EIS) were performed on an AUTOLAB PGSTAT302N workstation. Batteries rested at open-circuit voltage before testing, with an operating range of 0.01 V to 2 V at room temperature.

## 3. Results

### 3.1. Preparation Route and Morphological Characterization of BFAC@MEG

**Figure 1:**
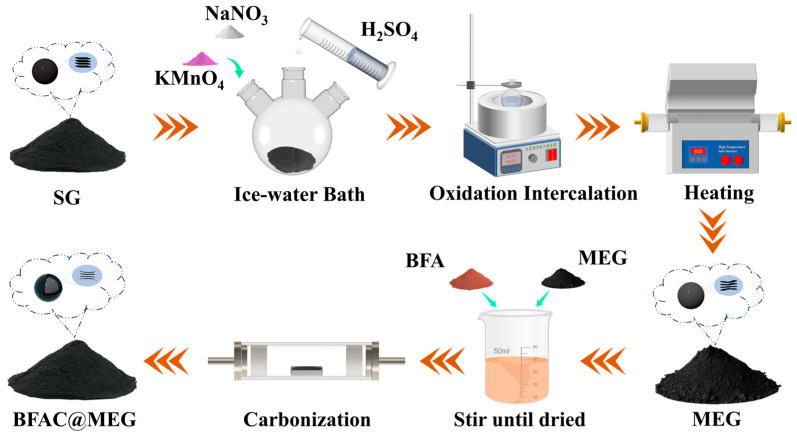
Schematic of the preparation process for MEG and BFAC@MEG.

Figure 1 illustrates a schematic representation of the preparation process for MEG and BFAC@MEG. SG displayed a stable and uniform structure of graphite layers. Through the combined action of KMnO_4_ and H_2_SO_4_, SG underwent oxidation and was subsequently subjected to a high-temperature environment to facilitate slight expansion, leading to the incomplete exposure of the edges of the graphite layers. The resultant product was designated as MEG. BFA is a cost-effective, water-soluble, small-molecule organic acid that can be effectively dispersed in an ethanol solution and combined with MEG [24]. XPS and EDS analyses reveal that BFA has high levels of N and S, which enhance the material’s rate performance. After mixing MEG with BFA evenly and drying, the mixture was transferred to a high-temperature environment for the carbonization treatment. The amorphous carbon derived from BFA was uniformly coated on the surface of MEG, and the resultant product was designated BFAC@MEG.

**Figure 2:**
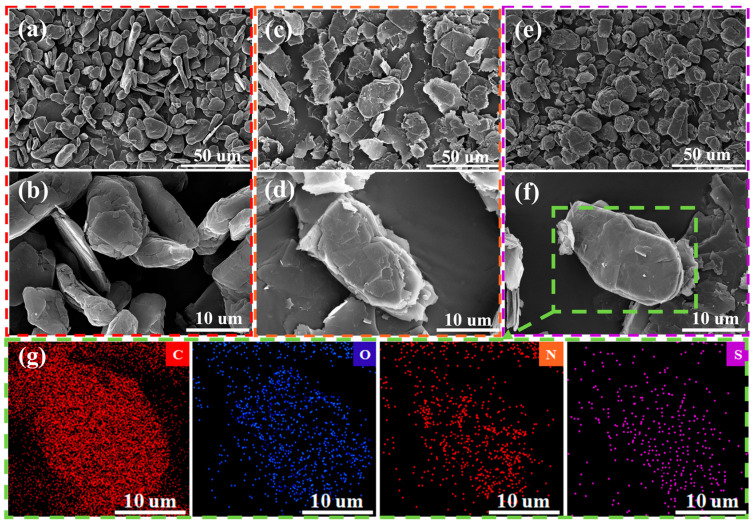
(**a**,**b**) SEM images of SG; (**c**,**d**) SEM images of MEG; (**e**,**f**) SEM images of BFAC@MEG; and (**g**) EDS elemental mapping of BFAC@MEG.

Figure 2a,b shows the SEM images of SG. Figure 2a reveals that SG has a consistent spherical structure with a particle size of about 17 µm. Figure 2b shows the SEM image of a single SG. Upon examination of the figure, it is apparent that not all SGs possess a complete spherical-like structure. The surface of SG is not entirely smooth as it still contains many broken graphite flakes and micropores. This situation might be responsible for an increased irreversible attrition of Li^+^ throughout the first charge–discharge cycle of the battery. Figure 2c,d illustrates the micromorphology of the MEG after slight expansion. Figure 2c shows that after the micro-expansion treatment, the particle size of MEG slightly increased, but it is not significantly different from that of SG. Figure 2d shows that MEG can still maintain a spherical-like structure similar to that of SG. The application of micro-expansion treatment minimally enhances the distance between graphite edge layers, which may facilitate a broader channel for Li^+^ intercalation and deintercalation. This modification may contribute to enhanced rate performance in LIBs. However, the exposure of the edge layers augments the material’s specific surface area, inevitably leading to increased electrolyte consumption during the initial charge–discharge cycle [22]. Therefore, MEG was coated with amorphous carbon derived from BFA, as shown in Figure 2e,f. Figure 2e shows that BFAC@MEG possesses a size comparable to that of SG and MEG. Figure 2f indicates that after the carbonization process, BFAC was uniformly coated on the MEG, making the surface of BFAC@MEG smoother. It is worth noting that the BFAC coating not only smooths the surface of MEG but also, due to the addition of the amorphous carbon layer, may reduce the interfacial charge transfer resistance, thereby promoting rapid ion storage kinetics [24]. In addition, the elemental distribution results showed that N and S were evenly dispersed across the surface of BFAC@MEG, as shown in Figure 2g. Figure S1 provides a detailed illustration of the EDS spectrum of BFAC@MEG, along with the elemental composition.

### 3.2. Physicochemical Properties of BFAC@MEG

**Figure 3:**
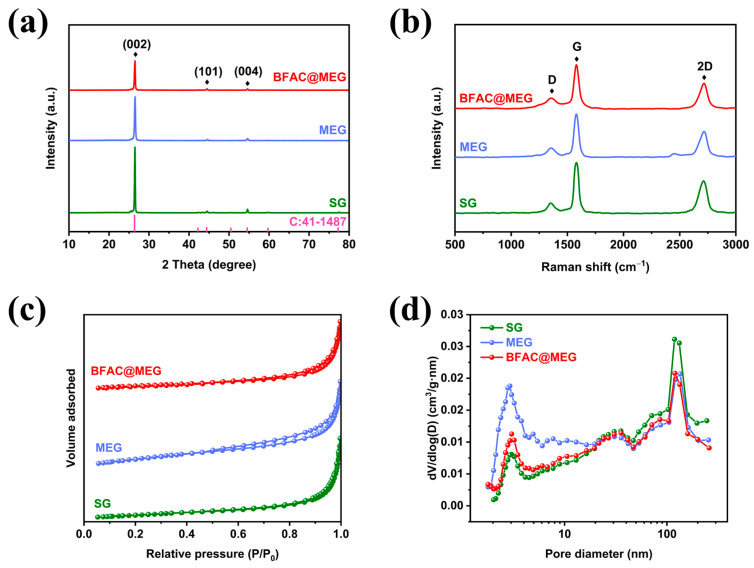
Characterization of the BFAC@MEG, MEG, and SG: (**a**) XRD patterns; (**b**) Raman spectroscopy analysis; (**c**) BET tests; and (**d**) the pore size distribution profiles.

The XRD data in Figure 3a offers a comparative insight into the interlayer spacing and degree of crystallinity present in the three materials. The XRD pattern of SG features three distinct diffraction peaks located at approximately 26.5°, 44.5°, and 53°, which are precisely aligned with the (002), (101), and (004) crystal planes of SG (PDF#41-1487) [29]. These graphite characteristic peaks are also present in the XRD patterns of MEG and BFAC@MEG. The intensity of the graphite (002) characteristic peak of MEG was significantly lower than that of SG, which may be due to the opening of the edge layers, resulting in a reduction in the level of graphitization of MEG. The intensity of the (002) peak of BFAC@MEG was further reduced compared to that of MEG. This diminishment can be traced to the incorporation of amorphous carbon during encapsulation, which subsequently lowers the material’s graphitization extent [18]. As depicted in Figure S2, the characteristic (002) diffraction peaks of BFAC@MEG and MEG exhibit a leftward shift relative to those of SG, with the most significant displacement observed for BFAC@MEG. Utilizing the positions of the (002) diffraction peaks for the three materials and applying the Bragg equation (*d_(002)_* = *λ*/2*sinθ_(002)_*), the corresponding interlayer spacings were determined and are summarized in Table S1. The interlayer spacings of SG, MEG, and BFAC@MEG are found to be 0.3360, 0.3362, and 0.3364 nm, respectively.

Raman testing can further demonstrate the variations in the degree of graphitization among the three samples. As shown in Figure 3b, BFAC@MEG, MEG, and SG exhibit three different peaks in the 500–3000 cm^−1^ range. The Raman peaks near 1350 cm^−1^ (*D*-peak) indicate structural defects and disorder in the graphite lattice. The *G*-peak is observed at approximately 1580 cm^−1^, while the *2D*-peak appears near 2680 cm^−1^ [30]. The ratio of the intensity of the *D* peak to that of the *G* peak (*I_D_*/*I_G_*) serves as an indicator of the material’s graphitic nature [26]. The *I_D_*/*I_G_* ratio is a well-recognized measure of a material’s disorder level, with higher values indicating more significant structural disorder. In this investigation, the *I_D_*/*I_G_* ratios were determined to be 0.24 for SG, 0.26 for MEG, and 0.28 for BFAC@MEG. These values suggest that the slight expansion-induced opening of graphite edge layers results in an increased number of defects and a higher degree of disorder within the graphite structure. Furthermore, the application of BFAC coating on MEG leads to a further increase in material disorder. As depicted in Figure 3b, all three materials exhibit strong *G* peaks, while the *2D* peaks are broad and asymmetric. These characteristics are indicative of layered graphite, suggesting that neither the micro-expansion treatment nor the carbon coating has disrupted the intrinsic layered structure of the graphite. The *I_2D_*/*I_G_* ratio is a crucial parameter for evaluating the number of layers, defect density, and overall structural quality of materials [31]. The introduction of defects during the micro-expansion treatment of SG results in an enhanced *D* peak intensity and a concomitant decrease in the relative intensity of the *2D* peak. Similarly, carbon coating of MEG leads to a reduction in the intensity of the *2D* peak. The calculated *I_2D_*/*I_G_* ratios for SG, MEG, and BFAC@MEG are 0.66, 0.62, and 0.60, respectively. These findings indicate that both micro-expansion treatment and carbon coating have increased the number of defects and the degree of disorder in the materials. The full width at half maximum (FWHM) of the *2D* peak provides additional evidence supporting these observations. The FWHM of the *2D* peak is closely correlated with the crystallinity of the material: higher crystallinity is associated with a narrower FWHM [32]. The FWHM values calculated for SG, MEG, and BFAC@MEG are 83.45, 87.55, and 91.65 cm^−1^, respectively. These results further confirm that micro-expansion treatment and carbon coating have reduced the graphitization degree of the materials. Detailed Raman spectroscopy analysis parameters for SG, MEG, and BFAC@MEG are provided in Supplementary Table S2.

BET analysis was employed to assess the SSA and pore structure of BFAC@MEG, MEG, and SG. Figure 3c shows the nitrogen adsorption/desorption isotherms for BFAC@MEG, MEG, and SG. The specific surface area of BFAC@MEG, MEG, and SG was calculated by using the BET method, which we found to be 5.59, 8.89, and 4.81 m^2^/g, respectively. The adsorption–desorption curve of SG is a type III isotherm, reflecting its typical layered structure. Within the pressure range of P/P_0_ = 0.45–1.0, BFAC@MEG and MEG displayed typical Type IV isotherms along with H3-type hysteresis loops [33]. The increase in the specific surface area and the appearance of typical hysteresis loops for MEG and BFAC@MEG are attributed to the slight expansion that opens the interlayer spacing at the edges of the graphite. The pore size distribution profiles of the materials, as illustrated in Figure 3d, were obtained through the application of the BJH method. The average pore sizes of SG, MEG, and BFAC@MEG were calculated to be 17.09, 35.86, and 22.21 nm, respectively. The pore size distribution diagram indicated that BFAC@MEG possessed a hierarchical mesoporous–macroporous structure [34]. This structure facilitates rapid wetting and diffusion of the electrolyte within the material, thereby enhancing the rate performance of the materials [35].

**Figure 4:**
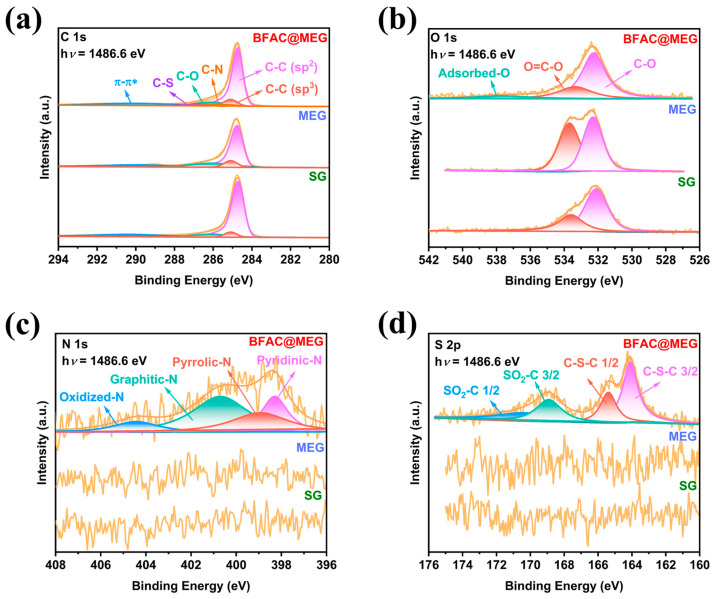
High-resolution XPS spectra of BFAC@MEG, MEG, and SG before electrochemical cycling: (**a**) C 1s; (**b**) O 1s; (**c**) N 1s; and (**d**) S 2p.

XPS analysis was used to examine the surface chemistry and elemental makeup of BFAC@MEG, MEG, and SG. Figure S3 shows the XPS survey spectra at h*ν* = 1486.6 eV for three different materials, highlighting the distinct differences among them. The presence of distinct N 1s and S 2p peaks in the XPS spectrum of BFAC@MEG indicates the successful incorporation of N and S into the material’s structure. Table S3 shows a detailed overview of the elemental composition and proportions of the three materials prior to electrochemical cycling. Figure 4a presents the C 1s spectra of BFAC@MEG, MEG, and SG. The C 1s peak was calibrated to a binding energy of 284.8 eV, and deconvolution analysis was performed on the components, yielding a series of fitted peaks. In accordance with the Doniach–Sunjic function, the peak at 284.7 eV in all three materials corresponds to the sp^2^-hybridized graphite C-C phase, which exhibits an asymmetric profile [36]. The π-π* satellite peak at 290.1 eV further corroborates the sp^2^-hybridized characteristic of these materials [37]. Apart from these peaks, the other characteristic peaks in the C 1s spectra of the three materials display symmetric shapes. Specifically, the peak at 285.1 eV is attributed to the sp^3^-hybridized C-C phase. The C-O bond is observed at 286.1 eV in all three materials. Unlike MEG and SG, the C 1s spectrum of BFAC@MEG features additional C-N and C-S components, located at 285.6 and 286.7 eV, respectively [36]. The relative intensity data (determined by peak area) of all components in the C 1s spectra of the three materials before electrochemical cycling are summarized in Table S4. In BFAC@MEG, the contributions of the C-N and C-S components are 3.50 and 3.42 at%, respectively.

Figure 4b displays the O 1s spectra for BFAC@MEG, MEG, and SG. The surfaces of BFAC@MEG, MEG, and SG all exhibit the presence of C-O and O=C-O groups, with binding energies at 532.2 and 533.5 eV, respectively [38]. Distinct from MEG and SG, BFAC@MEG exhibits a characteristic peak at 537.7 eV, which is attributed to adsorbed oxygen on the material surface. Figure 4c displays the N 1s spectra of the three materials. Notably, no N signal is detected on the surfaces of MEG and SG. In contrast, the N components on the surface of BFAC@MEG can be categorized into four types. The peak at 404.4 eV corresponds to oxidized N, while the peak representing graphitic N appears at 400.7 eV. The two peaks at binding energies of 399.3 and 398.3 eV are assigned to pyrrolic N and pyridinic N, respectively [24]. The incorporation of graphitic N notably boosts the material’s conductivity, which in turn enhances the rate performance of the LIBs anode. Figure 4d shows the S 2p spectra of the three materials. The two peaks at 164.1 and 165.4 eV are associated with the S 2p_3/2_ and S 2p_1/2_ peaks of the C-S-C covalent bond, confirming the successful doping of sulfur into BFAC@MEG [39]. Additionally, the two peaks near 168.9 and 170.3 eV correspond to the formation of sulfone (SO_2_-C _3/2_ and SO_2_-C _1/2_) [40].

**Figure 5:**
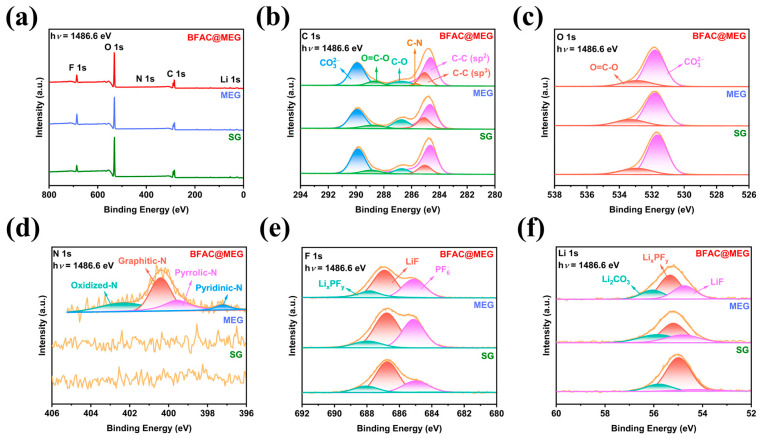
XPS spectra of BFAC@MEG, MEG, and SG electrodes after 500 cycles at 3 C: (**a**) survey spectra; (**b**) high-resolution C 1s spectra; (**c**) high-resolution O 1s spectra; (**d**) high-resolution N 1s spectra; (**e**) high-resolution F 1s spectra; and (**f**) high-resolution Li 1s spectra.

After 500 cycles at 3 C, the compositions and surface chemical states of the BFAC@MEG, MEG, and SG electrodes were further examined via XPS. As illustrated in Figure 5a, distinct XPS peaks for F 1s, O 1s, C 1s, and Li 1s are present in all three electrodes, while a faint N 1s signal is observed in the BFAC@MEG electrode. Table S5 presents the elemental composition on the surface of the three materials following electrochemical cycling. As indicated in Table S5, the atomic percentage of C on the surface of all three materials is approximately 31.5 at% after cycling, which represents a significant decrease compared to the pre-cycling levels (exceeding 90 at%). The detection of F and Li are attributed to the decomposition of the electrolyte on the material surfaces. In the case of BFAC@MEG, the surface atomic percentages of F and Li are 6.97 and 27.23 at%, respectively. Notably, the surface N content of BFAC@MEG drops to 0.60 at% after cycling, a marked reduction from the pre-cycling value of 1.08 at% (Table S3). Figure 5b presents the C 1s spectra of BFAC@MEG, MEG, and SG after the cycling process. In these spectra, an asymmetric C-C (sp^2^) peak is consistently observed at 284.7 eV for all three materials. The remaining characteristic peaks in the spectra are symmetric. The peak at 285.1 eV is attributed to the C-C (sp^3^) bond [36]. In the range of 286–289 eV, all three materials exhibit two peaks which are attributed to C-O (286.6 eV) and O=C-O (288.5 eV) [41]. The peak at 289.9 eV is associated with carbonate species on the material surface (${CO}_{3}^{2-}$), likely corresponding to Li_2_CO_3_ [39]. Table S6 presents a detailed analysis of the relative intensity data for each component in the C 1s spectra of the three materials. Specifically, the atomic percentage of the C-N component in BFAC@MEG is 1.13 at%. The atomic percentages of Li_2_CO_3_ in SG, MEG, and BFAC@MEG are 28.08, 25.56, and 29.26 at%, respectively. This indicates a greater abundance of Li_2_CO_3_ in the SEI film of BFAC@MEG. Li_2_CO_3_ is known for its high ionic conductivity, which facilitates the diffusion of Li^+^ within the material. This is a significant factor that contributes to the superior rate performance of BFAC@MEG compared to MEG and SG electrodes [42]. Additionally, a faint C-N peak at 285.6 eV is detected on the surface of BFAC@MEG.

Figure 5c presents the O 1s spectra of the three materials. The peaks at 531.8 and 533.5 eV are associated with ${CO}_{3}^{2-}$ and O=C-O, respectively. In the O 1s spectrum, the peak at 531.8 eV is most pronounced for BFAC@MEG, indicating that it has a higher concentration of Li_2_CO_3_ on its surface compared to the other materials [11]. Figure 5d illustrates the N 1s spectra of the three materials. N doping significantly enhances the nitrogen content on the surface of BFAC@MEG. However, following extensive electrochemical cycling, the N signal on the surface of BFAC@MEG weakened due to the formation of the SEI film. Specifically, the peak at 398.1 eV is indicative of pyridinic N, the one at 399.5 eV is ascribed to pyrrolic N, the peak at 400.6 eV is a marker for graphitic N, and the peak at 402.9 eV is characteristic of oxidized N [39]. Figure 5e displays the F 1s spectra of the three materials. The F 1s spectra show three peaks at energies of 685, 686.8, and 688 eV, which are associated with ${PF}_{6}^{-}$, LiF, and the inorganic parts of Li_x_PF_y_, respectively [43]. These inorganic components are formed during the SEI formation process. The presence of peaks at the same positions in the spectra indicates that the SEI compositions of the three materials are similar. However, MEG exhibited a higher LiF content than BFAC@MEG and SG, indicating more extensive decomposition of lithium salts on its surface. This is attributed to MEG’s larger specific surface area, which leads to more SEI formation during charge–discharge processes. Figure 5f shows the Li 1s spectra for the three materials. The peaks observed at 54.5, 55.1, and 56 eV correspond to LiF, Li_x_PF_y_, and Li_2_CO_3_, respectively. These assignments are in agreement with the observations from other spectra [11]. The XPS results demonstrate that all three materials have stable SEI films on their surfaces. The differences in specific surface area leads to varying extents of lithium salt decomposition on the surfaces of the three materials, resulting in different concentrations of SEI components.

**Figure 6:**
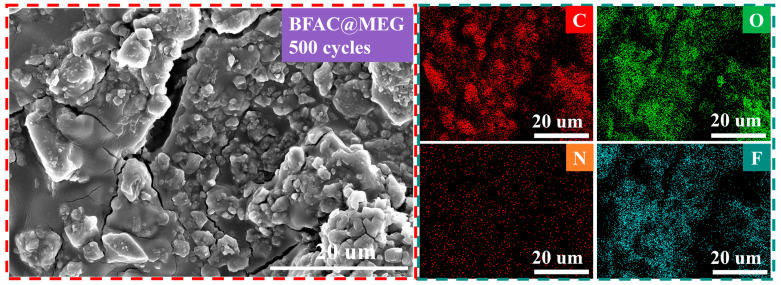
SEM and EDS elemental analysis of BFAC@MEG after 500 charge–discharge cycles at 3 C.

The surface morphology and elemental distribution of the electrode materials can be intuitively observed through SEM and EDS characterization of the electrode sheets after 500 cycles at 3 C. Figure 6 presents the morphology and elemental distribution of the BFAC@MEG electrode sheet. As depicted in the figure, following 500 charge–discharge cycles at 3 C cracks emerge on the electrode sheet. This phenomenon is primarily due to the volume expansion resulting from the repeated charge–discharge process. EDS analysis indicates that C, O, N, and F are evenly distributed on the surface of BFAC@MEG after cycling. For more detailed EDS spectral information of BFAC@MEG after cycling, refer to Figure S4.

To accurately determine the amount of amorphous carbon coating in BFAC@MEG, thermogravimetric analysis (TGA) was conducted on both BFAC@MEG and pure MEG. As shown in Figure S5, the TGA curves of BFAC@MEG and MEG were recorded within the temperature range of 30–800 °C. BFAC@MEG experienced a mass loss of about 1.1% between 30 and 600 °C, which is mainly attributed to the evaporation of moisture and the combustion of volatile constituents in the material. In contrast, MEG showed no significant mass loss between 600 and 670 °C, whereas BFAC@MEG experienced a mass loss of about 9% in this temperature range. Notably, MEG began to lose mass only when the temperature exceeded 670 °C. These observations suggest that the mass loss of BFAC@MEG between 600 and 670 °C can be attributed to the decomposition of the amorphous carbon coating on its surface [44]. Therefore, the amount of amorphous carbon coating in BFAC@MEG is determined to be 9%.

### 3.3. Electrochemical Performance of BFAC@MEG Anode

**Figure 7:**
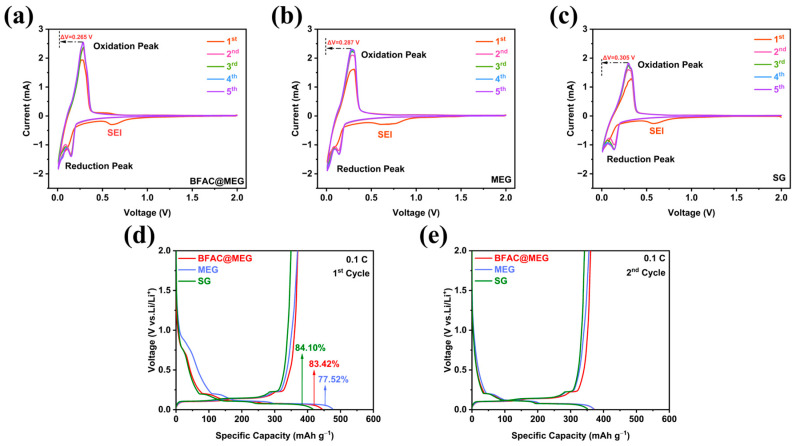
(**a**–**c**) 0.5 mV s^−1^ same scan rate CV curves of BFAC@MEG, MEG, and SG; (**d**) the first charge–discharge curves and ICE of BFAC@MEG, MEG, and SG; and (**e**) the second charge–discharge curves of BFAC@MEG, MEG, and SG.

Cyclic voltammetry (CV) was employed to examine the redox reactions involved during charge–discharge processes. The CV curves of BFAC@MEG, MEG, and SG are shown in Figure 7a–c. During the first cycles of the three materials, a prominent irreversible cathodic peak emerges near 0.65 V. In the second cycle, this peak vanishes, indicating an irreversible process [45]. The robust SEI layer forms during the initial charge–discharge cycle due to the interaction between the electrolyte and the electrode material. The reduction peak at around 0.15 V signifies Li^+^ intercalation, and the oxidation peak at about 0.30 V indicates Li^+^ deintercalation. A comparison of the voltage hysteresis in Figure 7a–c reveals that BFAC@MEG has the narrowest gap at 0.265 V, less than that of SG (0.305 V) and MEG (0.287 V), suggesting that BFAC@MEG undergoes the least electrochemical polarization in electrochemical cycles. The augmented electrochemical performance of BFAC@MEG can be ascribed to the integration of N and S, leading to a marked increase in the material’s electrical conductivity [24]. Figure 7d,e presents the first and second charge–discharge curves of BFAC@MEG, MEG, and SG. It is evident from the figure that the characteristic graphite plateau of SG during the charge–discharge process is well preserved in the BFAC@MEG composite anode [46]. Figure S6a,b indicates that between 0.5 and 1.0 V all three materials exhibit an electrochemical plateau during the first discharge process, with notable differences; however, this plateau disappears during the second discharge process. This phenomenon indicates the irreversible consumption of lithium and the formation of the SEI. This result is consistent with the behaviors observed in the CV curves of the three materials shown in Figure 7a–c.

A systematic comparison of the ICE changes during the first charge–discharge process for BFAC@MEG, MEG, and SG anode materials is presented. As shown in Figure 7d, based on the GCD curves, at 0.1 C (1 C = 372 mA g^−1^) the initial charge–discharge specific capacities for BFAC@MEG, MEG, and SG are 370/444, 369/476, and 352/419 mAh g^−1^. Upon calculation, at 0.1 C the charge capacities of BFAC@MEG and MEG were 18 mAh g^−1^ and 17 mAh g^−1^ higher than that of SG, respectively. This enhancement can be attributed to the slightly expanded structure of the electrodes, which provides more spacious pathways for lithium-ion intercalation and deintercalation, enabling deeper penetration of Li^+^ into the graphite matrix and thus increasing the available storage space for Li^+^. Additionally, the ICE of BFAC@MEG, MEG, and SG were determined to be 83.42%, 77.52%, and 84.10%, respectively. MEG has a lower ICE compared to SG, which can be attributed to the slight expansion that exposes the graphite edge layers. This increases MEG’s specific surface area, leading to a greater depletion of the electrolyte. Upon applying the BFAC coating to MEG there was a noticeable increase in the ICE, suggesting that the BFAC layer effectively minimized the material’s specific surface area and consequently curtailing electrolyte depletion in the inaugural charge–discharge cycle [22].

**Figure 8:**
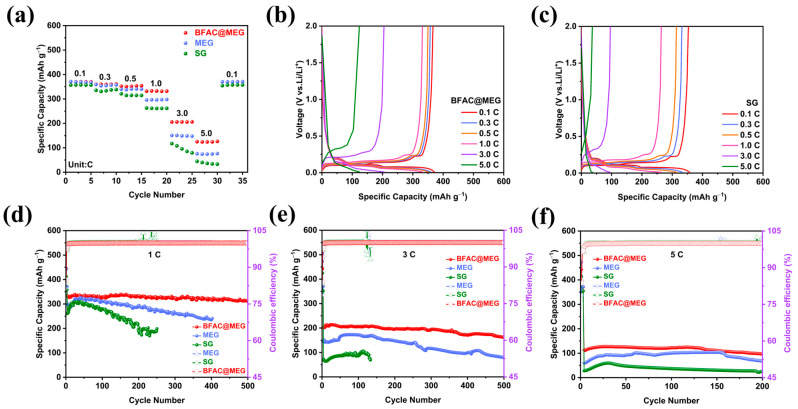
(**a**) Rate performance of BFAC@MEG, MEG, and SG; (**b**,**c**) charge–discharge curves of BFAC@MEG and SG at different rates; and (**d**–**f**) cycling curves of BFAC@MEG, MEG, and SG at 1 C, 3 C, and 5 C.

Figure 8a shows the rate performances of BFAC@MEG, MEG, and SG. As the rate increased from 0.1 C to 5 C, BFAC@MEG consistently exhibited higher reversible capacities than MEG and SG, with the differences becoming particularly pronounced at high rates. For instance, at 1 C BFAC@MEG had a specific capacity of 333 mAh g^−1^, which was 37 mAh g^−1^ higher than MEG and 72 mAh g^−1^ higher than SG. At 3 C, BFAC@MEG maintained a high specific capacity of 205 mAh g^−1^ while MEG and SG were both below 150 mAh g^−1^. Notably, at 5 C the specific capacity of BFAC@MEG was 1.68 times that of MEG and 3.44 times that of SG. Additionally, when the current rate decreased back to 0.1 C BFAC@MEG achieved a high reversible capacity of 370 mAh g^−1^, showing its superior rate capability and stability. These attributes can be attributed to the doping effects of N and S, as well as the addition of amorphous carbon layers. Table 1 presents the specific capacities of BFAC@MEG, MEG, and SG at various rates. Figure 8b presents the charge–discharge curves for the BFAC@MEG composite anode across various rates, spanning the voltage range of 0.01–2 V. From 0.1 C to 1 C there is almost no observation of the rise and shortening of the discharge platform. Even under the elevated rates of 3 C and 5 C the characteristic graphite platform of BFAC@MEG remained largely intact. However, Figure 8c indicates that the graphite signature platform of SG exhibits a pronounced increase and subsequent decrease with the acceleration of the rate. This suggests that the rate performance of SG is suboptimal. The results suggest that BFAC@MEG has the potential to serve as a high-rate anode material for LIBs. Table 2 presents some studies on natural graphite-based materials within the realm of high-rate LIBs. As shown in Table 2, compared with existing studies BFAC@MEG exhibits the highest high-rate capacity, especially at 3 C and 5 C.

To evaluate the cycling stability of BFAC@MEG, MEG, and SG, their cycling performance was tested at 1, 3, and 5 C rates. As shown in Figure 8d, at 1 C SG experienced a rapid capacity decline and MEG’s capacity dropped to less than 80% of its initial value after about 400 cycles. In contrast, BFAC@MEG remained stable after 500 cycles, retaining a significant reversible capacity of around 320 mAh g^−1^ with a retention rate of 95.92%. Figure 8e indicates that BFAC@MEG maintained 81.57% of its capacity at 3 C over 500 cycles. When the current rate reached 5 C, Figure 8f shows that BFAC@MEG maintained a high reversible capacity of 98 mAh g^−1^ after 200 cycles. Figure S7 indicates that at 5 C, BFAC@MEG exhibited a more pronounced graphite characteristic platform, a feature lacking in SG. Therefore, in contrast to the swift capacity decline observed in SG, the modified BFAC@MEG material demonstrated markedly enhanced reversible capacity, rate performance, and cycling stability.

**Figure 9:**
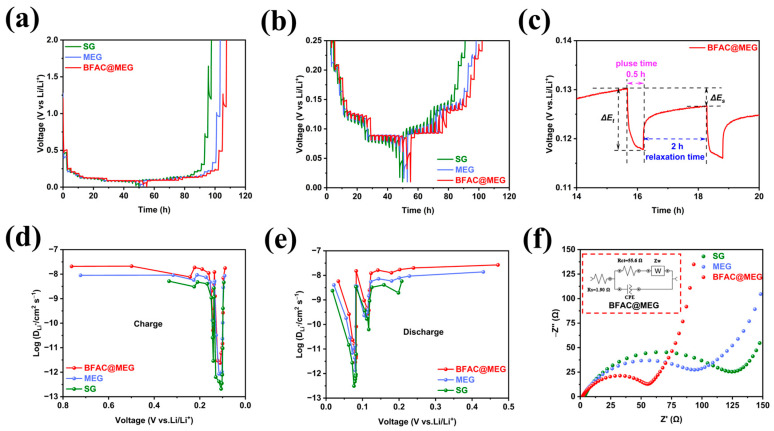
(**a**) GITT curves of BFAC@MEG, MEG, and SG; (**b**) local enlargement of GITT curves for the three materials; (**c**) enlarged view of a single cycle process; (**d**,**e**) comparison of lithium-ion diffusion coefficients for the three materials during charge and discharge processes; and (**f**) Nyquist plots and fitted circuit diagrams of BFAC@MEG, MEG, and SG before electrochemical cycling.

To understand the effects of BFAC addition on the electrochemical lithium storage mechanisms of the material, GITT was applied to scrutinize the Li^+^ diffusion dynamics within BFAC@MEG, MEG, and SG. The constant current intermittent titration curves of the three materials are presented in Figure 9a, with each charge–discharge cycle lasting 0.5 h at 0.1 C interspersed with 2 h rest intervals. Figure 9b shows a local enlargement of the GITT curves of the three materials. It can be seen that SG has larger voltage fluctuations at the end of discharge and charge, whereas BFAC@MEG exhibits the smallest voltage change. By applying Fick’s second law, the diffusion coefficients of Li^+^ through the three materials are determined from the voltage alterations due to the pulses and charge–discharge activities [49]. The diffusion coefficient for lithium ions (*D_Li_^+^*) was determined by employing the following Equation (1):

(1) $$D_{{\mathrm{Li}}^{+}}=\frac{4}{\pi \tau }{\left(\frac{m_{B}V_{m}}{M_{B}S}\right)}^{2}{\left(\frac{{\Delta E}_{s}}{{\Delta E}_{\tau }}\right)}^{2}\;,$$

where the values of Δ*E_s_* and Δ*E_τ_* can be derived from the single-step titration curves depicted in Figure 9c [50]. Figure 9d,e shows a comparative analysis of the variations in Li^+^ diffusion coefficients for BFAC@MEG, MEG, and SG throughout the charge–discharge cycles. Previous studies have shown that the diffusion coefficient of lithium ions in graphite typically ranges from 5.9 × 10^−12^ to 0.4 × 10^−9^ cm^2^ s^−1^ [51]. The calculated lithium-ion diffusion coefficients are 0.92–5.79 × 10^−9^ cm^2^ s^−1^ for SG, 0.15–0.94 × 10^−8^ cm^2^ s^−1^ for MEG, and 0.26–2.67 × 10^−8^ cm^2^ s^−1^ for BFAC@MEG. The illustrated results indicate that BFAC@MEG exhibits a superior Li^+^ diffusion coefficient over MEG and SG, reflecting the more efficient Li^+^ diffusion in BFAC@MEG. The observed effect could be due to N and S doping, which may lower the resistance to Li^+^ movement into and out of the material, subsequently boosting its rate performance.

The EIS method was applied to evaluate the charge transfer efficiency of BFAC@MEG, MEG, and SG before electrochemical cycling. As shown in Figure 9f, BFAC@MEG has the smallest semicircle arc diameter, indicating its superior charge transfer rate. Figure S8 is the Nyquist plots of the three materials in the low-frequency region. When contrasted with MEG and SG, BFAC@MEG displays the greatest linear slope at low frequencies, indicating a smaller Warburg impedance (*Z_w_*) and better facilitation of Li^+^ intercalation and deintercalation within the material. The fitted circuit diagram for the BFAC@MEG is shown in Figure 9f. By fitting the circuit for BFAC@MEG, MEG, and SG, a series of electrochemical AC impedance data were obtained in Table S7, with *R_s_* for BFAC@MEG, MEG, and SG being 1.90, 1.96, and 3.39 Ω, respectively, and *R_ct_* being 55.6, 103, and 123 Ω, respectively. BFAC@MEG has lower *R_s_* and *R_ct_* values than MEG and SG, proving that it is beneficial for enhancing the rate performance of the material.

### 3.4. The Influence of Electrochemical Cycling on the AC Impedance and SEI Film of Materials

**Figure 10:**
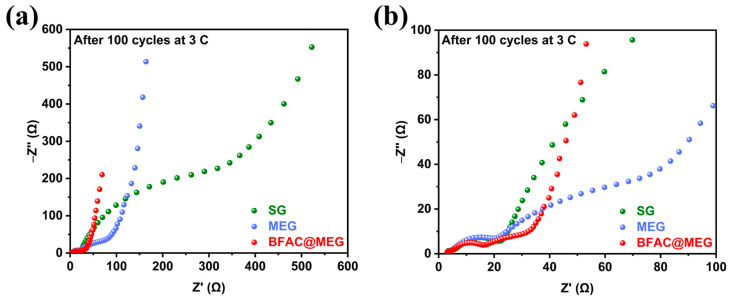
Nyquist plots of BFAC@MEG, MEG, and SG after 100 charge–discharge cycles at 3 C: (**a**) overall view; and (**b**) enlarged view of the high-frequency region.

The electrochemical impedance spectroscopy (EIS) test of cycled batteries can be used to determine the aging state of the battery and the thickness and uniformity of the SEI film. Figure 10a shows the Nyquist plots of BFAC@MEG, MEG, and SG after 100 charge–discharge cycles at 3 C. Based on this, the equivalent circuit fitting was carried out for the three materials (Figure S9). The figure clearly shows that the impedance of the cycled battery consists of four parts, namely the solution resistance (*R_s_*), the SEI film resistance (*R_SEI_*), the charge transfer resistance (*R_ct_*), and the Warburg impedance (*Z_w_*) [52]. The value of *Z_w_* can be ascertained by examining the slope of the straight line in the low-frequency region. A steeper slope of this line indicates a smaller *Z_w_*, which in turn suggests a stronger ion transfer capability of the material. As depicted in Figure 10a, following 100 cycles at 3 C, the slope of the straight line in the low-frequency region for SG has notably decreased, whereas the slope for BFAC@MEG remains the most substantial. This observation indicates that BFAC@MEG retains the strongest ion transfer ability even after cycling. Figure 10b illustrates the Nyquist plots of the three materials in the high-frequency region. Unlike their pre-cycling state, the Nyquist plots of these materials after electrochemical cycling display two semicircular arcs in the high-frequency region. The first semicircular arc primarily signifies the impedance of the SEI film (*R_SEI_*), whereas the second semicircular arc corresponds to the charge transfer impedance (*R_ct_*). The magnitude of *R_SEI_* can reflect the thickness and uniformity of the SEI film. A larger *R_SEI_* in the material suggests that the SEI film formed during the electrochemical cycling process is either thicker or less uniform, which consequently increases the transmission resistance of lithium ions. When the battery has poor cycling ability, the value of *R_ct_* will also increase sharply with the cycling of the battery [53]. A series of relevant impedance values can be obtained through equivalent circuit fitting, as shown in Table S8. The *R_s_* of SG, MEG, and BFAC@MEG are 5.77, 4.59, and 4.40 Ω, respectively. The *R_SEI_* are 19.0, 22.0, and 13.6 Ω, respectively. The *R_ct_* are 539, 117, and 46.8 Ω, respectively. It can be seen that after electrochemical cycling the *R_SEI_* of SG and MEG are much larger than that of BFAC@MEG, indicating that the SEI film on the surface of BFAC@MEG is the thinnest and most uniform. The *R_SEI_* of MEG is the largest because its specific surface area is the largest, and most of the SEI film is generated during the electrochemical reaction process. The *R_ct_* of SG is much larger than that of MEG and BFAC@MEG, indicating that SG ages the fastest during the electrochemical cycling process and has the worst cycling performance. The *R_ct_* of MEG and BFAC@MEG do not change significantly after 100 cycles, indicating that they have superior 3 C performance.

### 3.5. Analysis of the Structural Stability of Electrodes After Cycling

**Figure 11:**
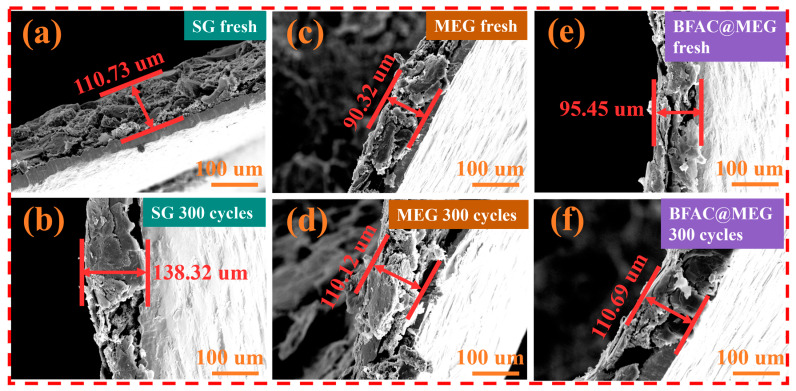
Cross-sectional SEM images of anode electrodes: (**a**) fresh SG electrode; (**b**) SG electrode after 300 cycles at 3 C; (**c**) fresh MEG electrode; (**d**) MEG electrode after 300 cycles at 3 C; (**e**) fresh BFAC@MEG electrode; and (**f**) BFAC@MEG electrode after 300 cycles at 3 C.

To investigate the influence of long-term charge–discharge cycling on the electrode structure, the initial and post-cycling states of SG, MEG, and BFAC@MEG electrodes were examined using cross-sectional SEM imaging. Figure 11a,b illustrates the cross-sectional morphology of the pristine SG electrode and the SG electrode after 300 cycles at 3 C, respectively. After performing calculations, it was found that the SG electrode showed a cross-sectional expansion of 24.92% after 300 charge–discharge cycles at 3 C. Likewise, Figure 11c,d illustrates the cross-sectional views of the original MEG electrode and the MEG electrode after undergoing 300 cycles at the same rate. As a result of the high-current charge–discharge process, the thickness of the MEG electrode rose from 90.32 μm to 110.12 μm, leading to a cross-sectional expansion of 21.92%. Compared to the SG electrode, the MEG electrode demonstrated a reduced cross-sectional expansion rate by 3.00%. This suggests that the edge-expanding structure of MEG helps to mitigate the compression and distortion of the graphite interlayer structure during lithium-ion intercalation and deintercalation, thereby alleviating the volume expansion of graphite caused by lithium-ion intercalation [11]. Figure 11e,f displays the cross-sectional images of the pristine BFAC@MEG electrode and the BFAC@MEG electrode after 300 cycles at 3 C. After 300 cycles at 3 C, the thickness of the BFAC@MEG electrode increased by 15.24 μm, leading to a cross-sectional expansion rate of 15.96%. Notably, the BFAC@MEG electrode exhibited the smallest cross-sectional expansion rate among the three electrodes, with values of 15.96%, 21.92% for MEG, and 24.92% for SG, respectively. This can be attributed to the protective shell formed by the BFAC coating on the surface of MEG, which possesses certain elasticity and flexibility. During lithium-ion intercalation and deintercalation, stress associated with volume expansion is generated within the graphite. The carbon coating layer effectively buffers these stresses, thereby significantly reducing the volume expansion of the material [54].

## 4. Conclusions

In summary, a micro-expanded graphite (MEG) with edge expansion was successfully synthesized, and by coating MEG with amorphous carbon derived from BFA, a N/S co-doped composite anode material (BFAC@MEG) was fabricated. The structure of MEG is conducive to the swift penetration of Li^+^ into the electrode material. The subsequent coating of MEG with BFAC results in a smoother surface for BFAC@MEG, and the incorporation of N and S enhances the electron and ion transport properties of the material, thereby significantly augmenting its capability for Li^+^ intercalation/deintercalation. GITT and EIS results indicate that the Li^+^ diffusion rate and charge transfer capability of BFAC@MEG are superior to those of MEG and SG. BFAC@MEG demonstrates relatively high-rate performance and cycling stability. At 1, 3, and 5 C the specific capacities of BFAC@MEG are 333, 205, and 124 mAh g^−1^, respectively. During the cycling tests, BFAC@MEG maintains an exceptional capacity retention of 95.92% after 500 cycles at 1 C. At 3 C BFAC@MEG retains 81.57% of its specific capacity after 500 cycles. Furthermore, at 5 C BFAC@MEG sustains a substantial reversible capacity of 98 mAh g^−1^ over 200 cycles. Morphological characterization and XPS analysis of the electrodes after cycling revealed that the BFAC@MEG electrode exhibited the smallest cross-sectional expansion rate. After 300 cycles at 3 C its expansion rate was only 15.96%, which was 5.96% and 8.96% lower than that of MEG and SG, respectively. The XPS results indicated that all three electrodes had stable SEI films on their surfaces after cycling, which were rich in inorganic components such as LiF and Li_2_CO_3_. This research confirms that the treatments of microporous expansion and N/S co-doping are advantageous for improving the rate capabilities of graphite anodes in LIBs, thereby expanding the avenues for the fabrication of modified natural graphite.

## Figures and Tables

**Table 1:** Specific capacities of three materials at 0.1–5 C in this work.

Materials	Specific Capacity (mAh g^−1^)					
	0.1 C	0.3 C	0.5 C	1 C	3 C	5 C
BFAC@MEG	370	359	351	333	205	124
MEG	369	355	340	296	149	74
SG	352	331	314	261	95	36

**Table 2:** Comparative analysis of performance for different natural graphite-based composite anodes.

	Materials	Specific Capacity (mAh g^−1^)							Ref.
		0.1 C	0.2 C	0.3 C	0.5 C	1 C	3 C	5 C	
1	BFAC@MEG	370		359	351	333	205	124	This work
2	MnO@MEG-13.2	450			350	270	100		[16]
3	NG-PN@C	330	325		200	52	20		[27]
4	P1G5	400	350		250	120		40	[29]
5	GMIB-2P	350	340		325	275		100	[39]
6	AG@HG@Al_2_O_3_		350		340	330	175	90	[47]
7	CMEG	361							[48]

## Data Availability

The original contributions presented in this study are included in the article/Supplementary Materials. Further inquiries can be directed to the corresponding author(s).

## Supplementary Materials

The following supporting information can be downloaded at: https://www.mdpi.com/article/10.3390/ma18112477/s1, Figure S1: EDS spectrum of BFAC@MEG before cycling; Figure S2: enlarged view of the characteristic (002) diffraction peaks of BFAC@MEG, MEG, and SG; Figure S3: XPS survey spectra of BFAC@MEG, MEG, and SG before electrochemical cycling; Figure S4: the EDS spectra of BFAC@MEG after 500 charge–discharge cycles at 3 C; Figure S5: thermogravimetric analysis (TGA) curves of BFAC@MEG and MEG from 30 to 800 °C; Figure S6: enlarged view of the discharge curves for the three materials between 0.5 and 1.0 V at 0.1 C: (a) first discharge cycle; and (b) second discharge cycle; Figure S7: charge–discharge curves of the three materials at 5 C; Figure S8: Nyquist plots of BFAC@MEG, MEG, and SG in the low-frequency region; Figure S9: the equivalent circuit diagram of the three materials after 100 cycles at 3 C; Table S1: the characteristic diffraction peaks of the (002) planes and the *d_(002)_* interlayer spacings for SG, MEG, and BFAC@MEG; Table S2: Raman spectroscopy analysis parameters of SG, MEG, and BFAC@MEG; Table S3: elemental composition and proportions of the three materials before electrochemical cycling; Table S4: relative intensities of the deconvoluted components in the C 1s spectra of the three samples before electrochemical cycling; Table S5: elemental composition and proportions of the three materials after electrochemical cycling; Table S6: relative intensities of the deconvoluted components in the C 1s spectra of the three samples after electrochemical cycling; Table S7: EIS data of SG, MEG, and BFAC@MEG before electrochemical cycling; and Table S8: EIS data of SG, MEG, and BFAC@MEG after 100 cycles at 3 C.
