# Supplementary material for: N/S Co-Doped Carbon-Coated Micro-Expanded Graphite for High-Performance Lithium-Ion Battery Anodes
Source: Materials (Basel). 2025 May 25;18(11):2477. doi: 10.3390/ma18112477 (PMC12155828; doi:10.3390/ma18112477)
Supplement: Supplementary file 1 [file materials-18-02477-s001.zip › materials-3608263-supplementary.pdf]

Supplementary Material for Materials

N/S Co-Doped Carbon-Coated Micro-Expanded Graphite for High-Performance Lithium-Ion Battery Anodes

Wenjie Wang [1,2], Xuan Zhang [3], Xianchao Wang [3], Chengwei Gao [4,*], Jinling Yin [1,2], Qing Wen [1,2,*] and Guiling Wang [1,2]

[1] Key Laboratory of Superlight Materials and Surface Technology of Ministry of Education, College of Materials Science and Chemical Engineering, Harbin Engineering University, Harbin 150001, China; wangwenjie914918@163.com (W.W.); yinjinling@hrbeu.edu.cn (J.Y.); wangguiling@hrbeu.edu.cn (G.W.)

[2] Heilongjiang Hachuan Carbon Materials Technology Co., Ltd., Jixi 158100, China

[3] CNBM Graphite New Material Co., Ltd., Beijing 100089, China; zhangxuan@cnbm.com.cn (X.Z.); wangxianchao@cnbm.com.cn (X.W.)

[4] Jixi Quality Supervision Inspection and Testing Center of Graphite Products (National Quality Inspection and Testing Center of Graphite Products (Heilongjiang)), Jixi 158100, China

* Correspondence: wenqing@hrbeu.edu.cn (Q.W.); gaochengwei1006@163.com (C.G.); Tel.: +86-13039978811 (Q.W.); +86-15845348882 (C.G.)

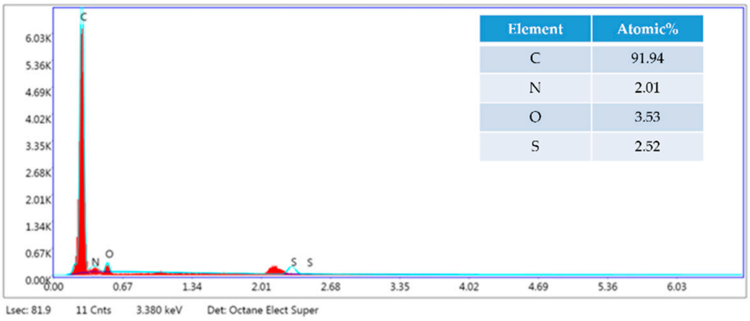

Figure S1. EDS spectrum of BFAC@MEG before cycling.

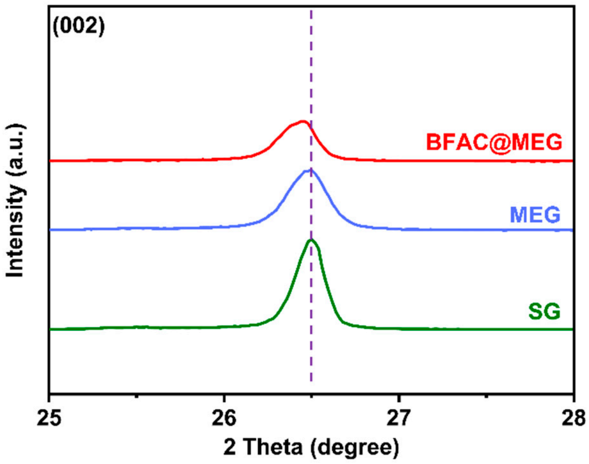

Figure S2. Enlarged view of the characteristic (002) diffraction peaks of BFAC@MEG, MEG and SG.

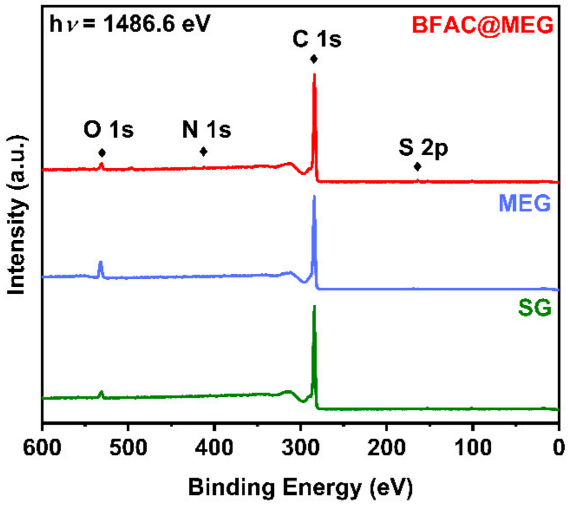

Figure S3. XPS survey spectra of BFAC@MEG, MEG and SG before electrochemical cycling.

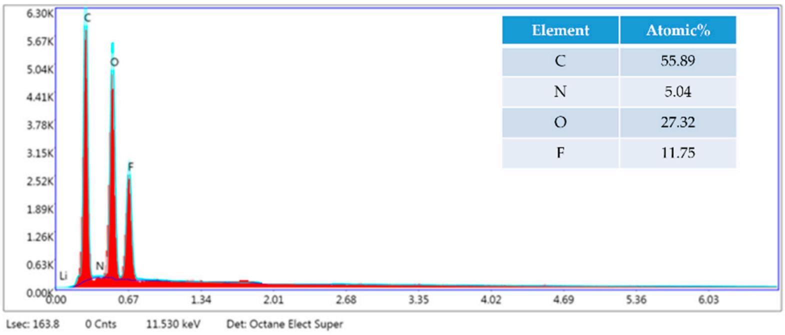

Figure S4. The EDS spectra of BFAC@MEG after 500 charge-discharge cycles at 3 C.

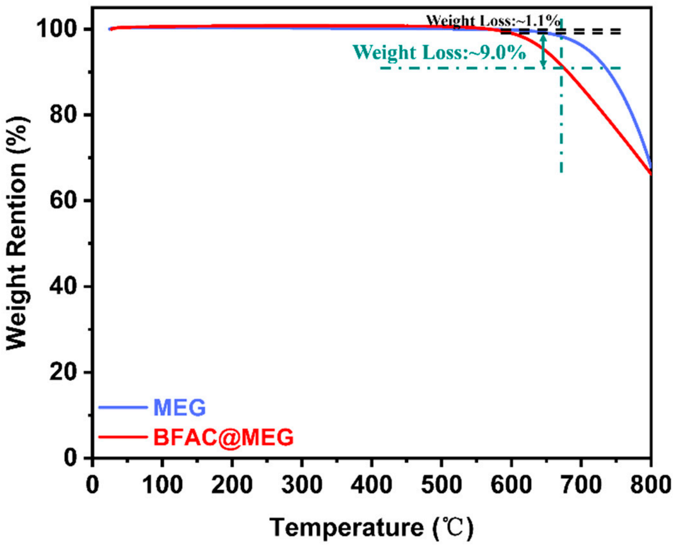

Figure S5. Thermogravimetric analysis (TGA) curves of BFAC@MEG and MEG from 30 to 800 °C.

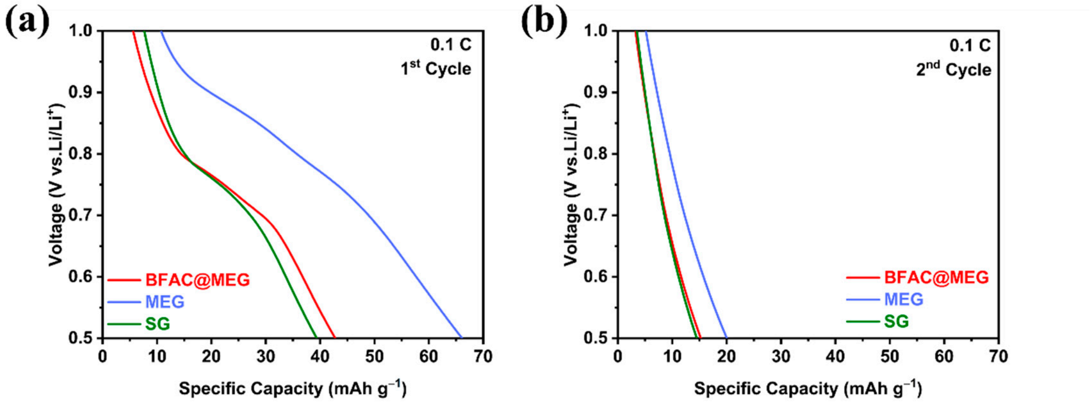

Figure S6. Enlarged view of the discharge curves for the three materials between 0.5 and 1.0 V at 0.1 C: (a) first discharge cycle; (b) second discharge cycle.

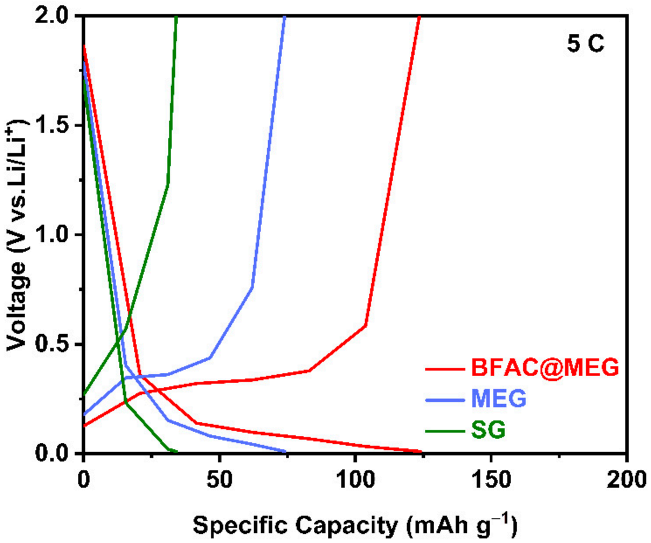

Figure S7. Charge-discharge curves of the three materials at 5 C.

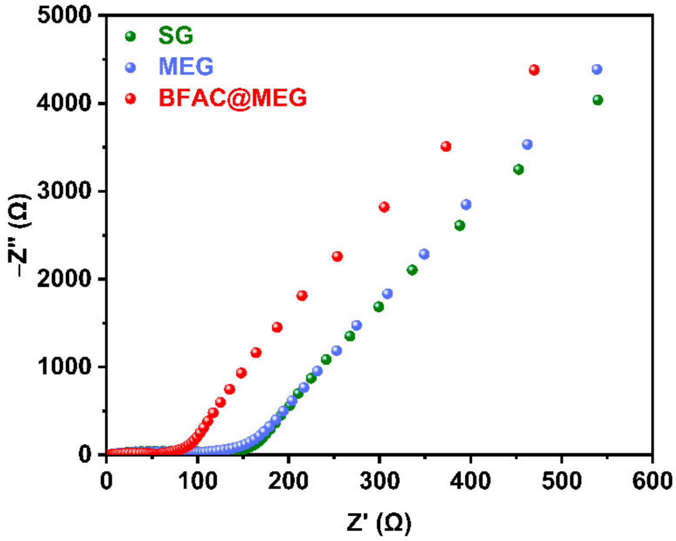

Figure S8. Nyquist plots of BFAC@MEG, MEG and SG in the low-frequency region.

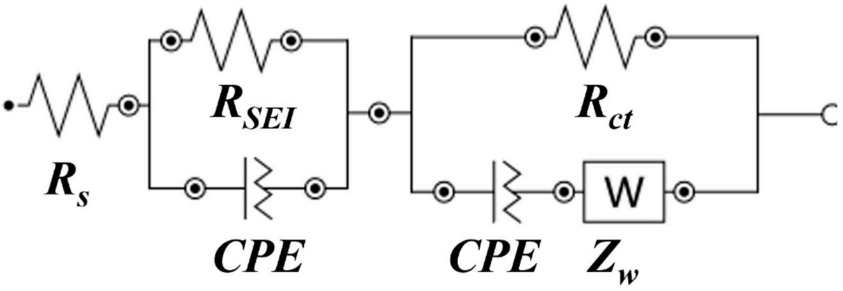

Figure S9. The equivalent circuit diagram of the three materials after 100 cycles at 3 C.

Table S1. The characteristic diffraction peaks of the (002) planes and the $d_{(002)}$ interlayer spacings for SG, MEG and BFAC@MEG.

Materials	SG	MEG	BFAC@MEG
2θ (°)	26.50	26.48	26.46
$d_{(002)}$ (nm)	0.3360	0.3362	0.3364

Table S2. Raman Spectroscopy Analysis Parameters of SG, MEG and BFAC@MEG.

Materials	I_D/I_G	I_{2D}/I_G	FWHM of *2D* (cm^{-1})
SG	0.24	0.66	83.45
MEG	0.26	0.62	87.55
BFAC@MEG	0.28	0.60	91.65

Table S3. Elemental composition and proportions of the three materials before electrochemical cycling.

Samples	C (at%)	O (at%)	N (at%)	S (at%)
SG	96.51	3.49	-	-
MEG	91.80	8.20	-	-
BFAC@MEG	95.12	2.37	1.08	1.43

Table S4. Relative intensities of the deconvoluted components in the C 1s spectra of the three samples before electrochemical cycling.

Samples	Peaks (Components (at%))				
	C-C (sp^2)	C-C (sp^3)	C-N	C-O	C-S
SG	84.97	5.72	-	9.31	-
MEG	74.41	8.79	-	16.80	-
BFAC@MEG	76.59	7.46	3.50	9.03	3.42

Table S5. Elemental composition and proportions of the three materials after electrochemical cycling.

Samples	C (at%)	O (at%)	N (at%)	F (at%)	Li (at%)
SG	31.53	35.48	-	5.35	27.64
MEG	31.55	33.21	-	9.85	25.39
BFAC@MEG	31.32	33.88	0.60	6.97	27.23

Table S6. Relative intensities of the deconvoluted components in the C 1s spectra of the three samples after electrochemical cycling.

Samples	Peaks (Components (at%))					
	C-C (sp^2)	C-C (sp^3)	C-N	C-O	O=C-O	CO_3^{2-}
SG	52.29	8.71	-	5.81	5.11	28.08
MEG	40.96	14.93	-	12.44	6.11	25.56
BFAC@MEG	44.52	12.38	1.13	7.32	5.39	29.26

Table S7. EIS data of SG, MEG and BFAC@MEG before electrochemical cycling.

Materials	R_s (Ω)	R_{ct} (Ω)
SG	3.39	123
MEG	1.96	103
BFAC@MEG	1.90	55.6

Table S8. EIS data of SG, MEG and BFAC@MEG after 100 cycles at 3 C.

**Materials**	R_s **(Ω)**	R_{SEI} **(Ω)**	R_{ct} **(Ω)**
SG	5.77	19.0	539
MEG	4.59	22.0	117
BFAC@MEG	4.40	13.6	46.8
